# Nonsurgical Repair of the Ascending Aorta: Why Less Is More

**DOI:** 10.3390/jcm12144771

**Published:** 2023-07-19

**Authors:** Xun Yuan, Xiaoxin Kan, Zhihui Dong, Xiao Yun Xu, Christoph A. Nienaber

**Affiliations:** 1 Cardiology and Aortic Centre, Royal Brompton & Harefield Hospitals, Guy’s and St Thomas’ NHS Foundation Trust, London SW3 6NP, UK; x.yuan@rbht.nhs.uk; 2 National Heart and Lung Institute, School of Medicine, Imperial College London, London SW3 6LY, UK; 3 Center for Vascular Surgery and Wound Care, Jinshan Hospital, Fudan University, Shanghai 201508, China; x.kan17@imperial.ac.uk (X.K.); dong.zhihui@zs-hospital.sh.cn (Z.D.); 4 Department of Chemical Engineering, Imperial College London, London SW7 2BX, UK; yun.xu@imperial.ac.uk

**Keywords:** ascending aorta, endovascular repair, stent graft, vascular occluder, false lumen, aortic remodelling, FLIRT

## Abstract

*Objective:* Advanced endovascular options for acute and chronic pathology of the ascending aorta are emerging; however, several problems with stent grafts placed in the ascending aorta have been identified in patients unsuitable for surgical repair, such as migration and erosion at aorta interface. *Method:* Among the six cases analysed in this report, three were treated with a stent graft in the ascending aorta to manage chronic dissection in the proximal aorta; dimensions of those stent grafts varied between 34 and 45 mm in diameter, and from 77 to 100 mm in length. Three patients, matched by age, sex and their nature of pathology, were subjected to the focal closure of a single communicating entry by the use of an occluding device (Amplatzer ASD and PFO occluders between 14 and 18 mm disc diameter) with similar Charlson comorbidity score. *Results:* Both conceptually different nonsurgical management strategies were technically feasible; however, with stent grafts, an early or delayed erosion to full re-dissection was documented with stent grafts, in contrast to complete seal, with an induced remodelling and a long-term survival after the successful placing of coils and occluder devices. Moreover, aortic root motion was not impaired by the focal occlusion of a communication with an occluder, while free motion was impeded after stent graft placement. *Conclusions:* The intriguing observation in our small series was that stent grafts placed in the ascending aorta portends the risk of an either early (post-procedural) or delayed migration and erosion of aortic tissues at the landing site or biological interface between 12 and 16 months after the procedure, a phenomenon not seen with the use of focal occluding devices up to 5 years of follow-up. Obviously, the focal approach avoids the erosion of the aortic wall as the result of minimal interaction with the biological interface, such as a diseased aortic wall. Potential explanations may be related to a reduced motion of the aortic root after the placement of stent graft in the ascending aorta, whereas the free motion of aortic root was preserved with an occluder. The causality of erosion may however not be fully understood, as besides the stiffness and radial force of the stent graft, other factors such as the induced inflammatory reactions of aortic tissue and local adhesions within the chest may also play a role. With stent grafts failing to portend long-term success, they may still have a role as a temporizing solution for elective surgical conversion. Larger datasets from registries are needed to further explore this evolving field of interventions to the ascending aorta.

## 1. Introduction

Advanced endovascular options for the acute and chronic pathology of the ascending aorta are emerging and have reached the clinical arena [1,2]. Observations in small case series and registries have identified several problems with stent grafts placed in the ascending aorta in patients who are not candidates for surgical repair, such as migration and erosion at the stent graft and aorta interface [3,4,5,6,7]. One of the reasons for those serious complications is related to the three-dimensional movement of the ascending aorta in the thoracic cage and the subsequent friction between the ends of a placed stent graft and the ascending aorta [4,8,9]. Although early success has been described in selected patients with focal aneurysmatic transformation or chronic localised dissection by virtue of sealing the entry to either false lumen or aneurysmatic space [10], longer-term observations have at best shown a temporizing effect when using stent graft in this area [11]. In the acute/subacute setting, case reports and the ARISE trial have failed to show a lasting positive effect [3,4].

While stent grafts placed into the ascending aorta have been associated with migration and erosion, various reports on the focal patching of entry tears using septal occluders or occluder-like instruments [5,6] have shown promise with no midterm erosion or migration [6]. In this paper, we test the hypothesis whether sealing an entry tear or communication between true and false lumen by an occluder device would lead to similar or better results than stent grafts placed in the ascending aorta in patients with focal aneurysmatic disease or chronic aortic dissection. For this pilot study, three consecutive patients who underwent endovascular stenting in the ascending aorta were compared to three patients subjected to focal entry closure by an occluding device, and followed over 5 years.

## 2. Methods

### 2.1. Study Design

Our analysis is based on a retrospective matched cohort (head-to-head) comparison of two methods to isolate the false lumen in inoperative patients with a proximal type of aortic dissection. All patients had a DeBakey type II pathology with a focal dissection in the ascending aorta.

### 2.2. Demographics

Patients who underwent nonsurgical repair for chronic pathology in the ascending aorta had been considered unsuitable for surgical repair, with the idea to seal the entry tear of communication to a false lumen in chronic type A dissection by an interventional procedure under general anaesthesia; none of the 6 patients were treated in the acute phase of dissection. Among all six cases analysed in this report, 3 patients were treated with a stent graft in the ascending aorta (with 2 males and 1 female patient) at an average age of 77.7 ± 1.53 years; dimensions of those stent grafts varied between 34 and 45 mm, while varying in length from 77 to 100 mm. Three patients matched by age, sex and nature of pathology were subjected to the focal closure of communicating entry by use of an occluding device (Amplatzer ASD and PFO occluders between 14 and 18 mm disc diameter); there were also 2 males and 1 female patient aged 79.3 ± 5.13 years (Table 1). The Charlson comorbidity score was high in both groups, ranging between 3 and 9 in the stent graft group versus 4 and 5 in the comparator.

### 2.3. Procedural Details

Technical and procedural details are individually listed in Table 1. Both with the placement of stent graft and with occluder deployment, a wide range of procedural time and radiation burden was documented; however, there was a trend towards a shorter hospital stay and use of resources with occluder devices than with stent grafts due to the fully percutaneous approach. Conversely, for TEVAR procedures in collaboration with vascular surgeons, access was established by surgical cutdown to the femoral artery. For the respective interventions, either commercially available stent grafts were used (Zenith^®^ TX2^®^ COOK^®^ Medical, GoreTag^®^ or CTag) or commercially available ASD and PFO occluders (Occlutech^®^ or Amplatzer™). Initial intraprocedural success was seen in all patients with early failure in 1 case after stent graft and late failure in 2 cases after stent graft, essentially using technology and techniques as previously published elsewhere [5,12]. 

CT images prior to TEVAR were reviewed by experienced radiologists and the size of the stent graft was chosen based on the measurement of pre-TEVAR CT images. The proximal sealing zone was determined at a level at least 2 cm apart from the entry in the dissection lamella. Stent graft dimensions were determined by the estimated true lumen diameter at proximal sealing segment. All TEVAR procedures were performed under general anaesthesia, allowing for vascular cutdown in the groin to expose the femoral artery for access. A pigtail catheter was inserted over a guide wire via a 6 French introducer, then exchanged to a stiff wire over pigtail catheter in the true lumen of the ascending aorta. Digital subtraction angiography (DSA) imaging via the pigtail catheter was used to check the dissection and confirm the location and proximal sealing zone. After exchange for DrySeal introducer, the delivery system for a proximal stent graft was inserted over a Lunderquist wire (Boston Scientific, Marlborough, MA, USA) and positioned carefully under fluoroscopy. To ensure the designated satisfactory landing position, the stent graft was deployed under rapid right ventricular pacing to reduce systolic blood pressure to 60 bpm during launch. DSA was repeated after launch of stent graft to document stent graft placement and sealing of the communication between true and false lumen. After the angiographic image acquisition, all instruments and introducers were removed, followed by a surgical repair of the femoral artery access.

Prior to the use of a focal ASD or PFO occluding device, similar with TEVAR, pre-procedural CT images were reviewed by experienced radiologists, and details of focal dissection, width and depth of false lumen, and the diameter of the dissected aorta were measured from appropriate CT angiographic images. The size of any given occluder was chosen based on both the diameter of the communication (or entry) and the depth of false lumen to accommodate the distal disc of an occluder device in the false lumen, thus determining the required dimension to seal the communication between true and false lumen. The waist of ADS/PFO occluder device was smaller than the diameter of the diameter of focal entry tear. Via percutaneous approach from a femoral artery, a coronary multi-purpose catheter was utilised to identify and navigate the focal dissection lesion and advance the tip into false lumen under fluoroscopy. As a first step, some coils were advanced via the multi-purpose catheter into the false lumen (to promote later thrombus formation); secondly, a normal exchange length of 0.035 inches of wire was advanced in the false lumen over the multi-purpose catheter, which was then removed in exchange for a delivery sheath for the occluder. Deployment of the distal umbrella in the false lumen followed by the deployment of the proximal umbrella in the true lumen was subsequently monitored by fluoroscopy and documented on a final DSA run to prove the exact placement and sealing of the communication.

### 2.4. Medication

All patients were treated simultaneously for underlying chronic arterial hypertension by a combination of at least three different drugs, assuring a low normal blood pressure; there were no obvious differences between groups (Table 2).

### 2.5. Follow-Up and Survival

A 5-year follow-up was documented for all six patients. Clinical surveillance was conducted in all patients over 5 years with annual clinical appointments, including echocardiographic assessment and contrast-enhanced CT imaging (Table 2).

### 2.6. Motion Analysis

For each patient, pre- and post-procedural DSA images were adopted to perform quantitative analysis of device-induced aortic motion alteration. The two-dimensional DSA images were acquired at a frame rate of 4 frames per second during an average scan time of 5.5 s (minimum 3 s). Hence, for an individual scan, a minimum of 12 frames were obtained and analysed. The open-sourced medical image analysis package 3D Slicer was adopted for marking the spatial position of anatomical landmarks on DSA images. 

**Figure 1:**
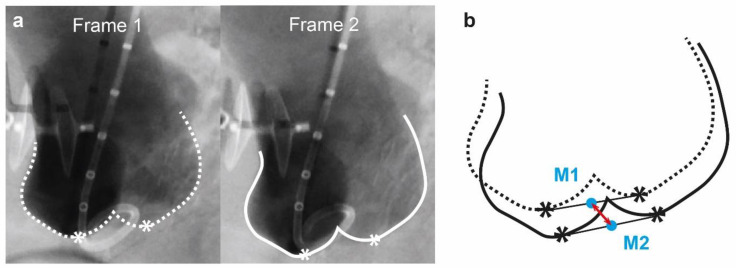
Illustration of the measurement of aortic root motion from DSA images. (**a**) For each angiographic frame, reference points at the base of aortic sinuses were marked. (**b**) The mid-points of the reference points were calculated and adopted to represent the position of aortic root in each frame. Maximum distance between the mid-points was measured as the maximum aortic root motion within the total scanning time.

DSA images were analysed frame by frame by following the methodology described in a previous study [13]. The base of 2 aortic sinuses shown on DSA images were marked as reference points, while the mid-point of the two reference points was used to represent the location of the aortic root in the current frame (Figure 1). After marking mid-points in all frames during the total scanning time, the maximum distance of all mid-points were calculated as the maximum motion range of aortic root by using MATLAD (MathWorks, Natick, MA, USA). The extent of aortic root motion before and after each intervention is listed in Table 3 and illustrated in Figure 1.

## 3. Results

**Figure 2:**
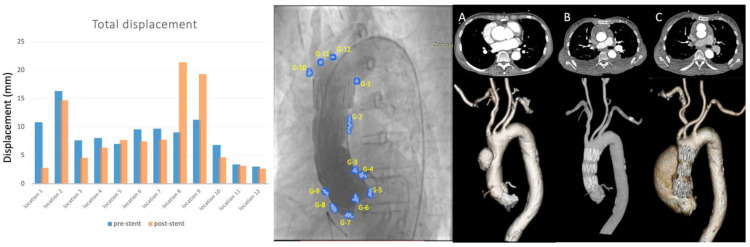
Aortic root motion for a patient treated with a stent graft in the ascending aorta. The composite illustration shows numeric values of displacement at each reference point before and after the placement of a stent graft on the left. The centre piece shows one given angiographic frame with attached reference points; and on the right, the reconstructed CT angiographic images are depicted before the endovascular intervention (**A**), with the stent graft in place (**B**); and finally, 16 months after the intervention (**C**), the creation of a re-dissection from a stent graft-induced erosion is revealed.

**Figure 3:**
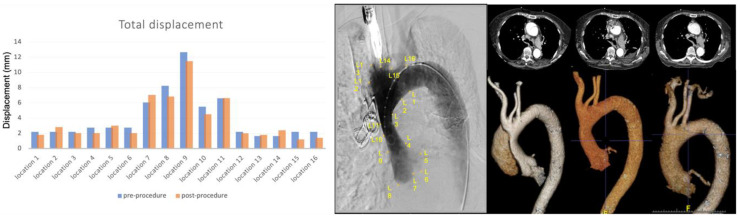
Aortic root motion in a patient treated with an ASD occlude and additional coils to seal localised ascending aortic dissection. The composite illustration shows the numeric values of displacement at each reference point before and after placement of an ASD occluder on the left. The centre piece shows one patient given an angiographic frame after coils were placed in the false lumen, and the complete occlusion of the entry tear by the use of double umbrella occluder; on the right, note the reconstructed CT angiographic images demonstrating the complete occlusion of any communication to the false lumen, successful remodelling over 3 months from before the endovascular intervention with no evidence of any remaining false lumen.

Both conceptually different nonsurgical management strategies are illustrated in typical case examples; Figure 2 shows a case of a stent graft placed in an ascending aorta while Figure 3 illustrates the focal sealing of an entry tear by the use of an Amplatzer™ occluder device in a similar setting of proximal communication in type A aortic dissection. Note the early erosion to full re-dissection in contrast to complete seal and induced remodelling after the placement of an occluder device.

Demographic and procedural details are summarised in Table 1. Group 1 (stent graft) was similar to group 2 (focal use of occluding device) with regard to age, gender distribution and nature of pathology. Patients in both groups were unsuitable for open surgical repair, considering their high comorbidity profile by the Charlton score and EuroScore II. The Charlson comorbidity index ranged from 3 to 9 in group 1, and 4 to 5 in group 2; and EuroScore II ranged from 4.88 to 20.4 in group I, compared with 11.47 to 31.41 in group 2. The total diameter of ascending aorta was similar in both groups. Every patient has one proximal entry tear in the aorta and could therefore be considered for DeBakey II aortic dissection. 

Procedural details were similar between groups in view of the use of general anaesthesia time, procedural duration and the amount of contrast dye used; there was a trend towards a higher radiation burden in cases undergoing interventional occluder placement (as a less standardised method). However, the patients receiving an occluder device enjoyed a shorter hospital stay due to the total percutaneous procedure with an approximal 4 ± 1 days compared to the stent graft group with 11 ± 6 days (Table 1). An immediate procedural success was seen in two of the three patients undergoing stent graft placement compared to the three cases undergoing interventional occluder placement. The hospital stay in group 1 was longer compared with group 2 (occluder devices), owing to surgical cutdown to the femoral artery for large bore access.

The post-intervention medication used in each patient and the follow-up outcomes are summarised in Table 2. The medication and combination of drugs were essentially similar between groups. While reinterventions were necessary after stent graft placement, such as conversion to open surgery, no reintervention was required in patients after the placement of an occluder device (group 2) over the entire follow-up period of 5 years with completed false lumen thrombosis and remodelling (Figure 3). In contrast, two cases developed stent-induced new entry tear (SINE), and one case was unsuccessful due to peri-procedural stent graft migration in group 1. The outcomes in terms of survival pattern reveal one death and one conversion to open surgery after stent grafting (group 1), while the mortality and reintervention rates in group 2 were zero over at least 5 years. Despite of the need for conversion to open surgery, group 1 patients survived at least 1 year with one death soon after 1 year. Figure 2 and Figure 3 display a typical example from each group, also highlighting the similarity of the pathologies treated.

**Figure 4:**
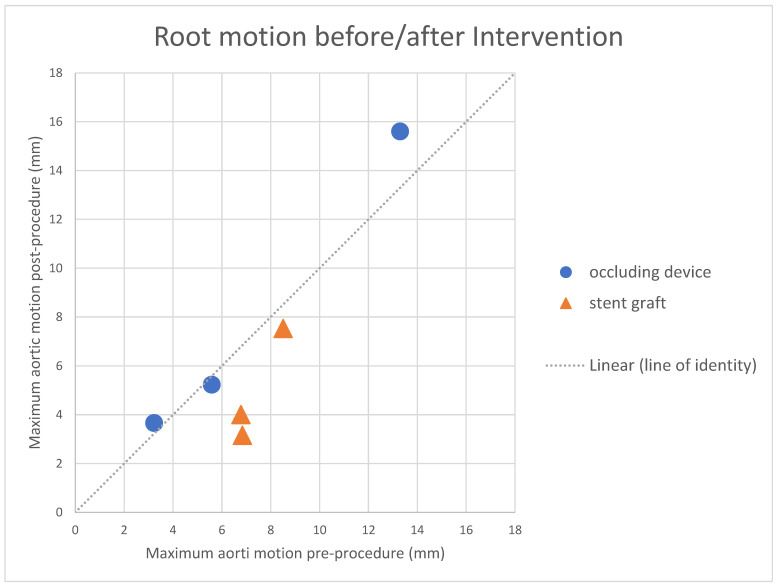
This graph illustrates the motion of the aortic root before and after the placement of either a stent graft or an occluder device; the ratio of motion (before and after each intervention) reveals that no patients fitted with an occluder revealed any significant impairment of motion as they are located on the line of identity (marked in blue). Conversely, with a stent graft placed in the ascending aorta, post-interventional aortic motion was impaired to varying extents in those three patients (in orange).

The extent of aortic root motion before and after either stent graft placement of occluder deployment is listed individually in Table 3 and summarised in Figure 4; the graphical display illustrates that aortic root motion was not impaired by the focal occlusion of a communication with an occluder and was similar before and after the intervention in all three cases on the line of identity. Conversely, with a stent graft, the free motion of the aortic root was found to be impeded with markedly less motion after stent graft placement than before in all three cases (Figure 4).

## 4. Discussion

Our comparison between two nonsurgical strategies to address the ascending aorta in selected patients unsuitable for open repair has shown that the focal closure and sealing of entry sites is technically feasible in the chronic dissection of the ascending aorta. Both the focal use of occluders and the short stent graft aim for the same goal: to depressurise the false lumen by closing entry tears, thereby initiating thrombosis and the remodelling of the false lumen in the setting of chronic ascending aortic dissection. While the concept of sealing entry tears by the use of stent grafts has been successfully shown in the descending aorta (e.g., in type B dissection) its application to pathologies in the ascending aorta is at best controversial [14,15]; the concept of the focal occlusion of entry tears by the use of occluders and coils as a primary strategy is new and limited in case reports [5,6]; there seems to be a consensus that only patients with a prohibitively high risk for open surgery may be candidates for any interventional approach in this setting, as in our observational series of six cases.

The intriguing observation in our small series was that stent grafts placed in the ascending aorta portend the risk of either an early (post-procedural) or a delayed migration and erosion of aortic tissues at the landing site or biological interface between 12 and 16 months, a phenomenon not seen with the use of focal occluding devices up to 5 years of follow-up. While all three patients after stent graft treatment required either immediate or late surgical conversion (with one death), patients selected for occluder devices (including coils) to seal entry points demonstrated remodelling and survived >5 years with no need for further intervention. Obviously, the focal approach avoids the migration and erosion of the aortic wall as the result of minimal interaction with the biological interface, e.g., the diseased aortic wall. Moreover, a complete seal and an induced thrombosis of the false lumen with subsequent remodelling were demonstrated in all three cases of ascending aortic pathology (Figure 3). While those observations in a small set of patients are quite interesting, explanations are not entirely clear yet and rather speculative, but conceptual differences in the approaches may provide at least some answers. Radial force may play a role in stent grafts and may impact the interface between stent grafts and native aortic walls which are likely to cause erosion, particularly with relatively rigid devices such as Zenith^®^ TX2^®^. 

Our analysis of aortic root motion in the chest before and after each intervention had clearly revealed some degree of a reduced motion of the aortic root after the placement of a stent graft in the ascending aorta, whereas the free motion of the root was preserved after sealing an entry with an occluder (Figure 4); this signal was consistent and clearly separated the two groups with regard to post-procedural aortic motion, and may play a predisposing role for aortic wall erosion observed after stent graft placement. The causality of erosion may however not be fully understood yet, as besides the stiffness of the stent graft, other factors such as the stent-induced inflammatory reactions of aortic tissue and local adhesions within the chest may also play a role. 

Conversely, with the use of a focal closure of an entry tear, the synchronic swinging motion of the aortic root remains uninhibited and may avoid the untoward consequences of stent grafts in the ascending aorta. In fact, the extent of aortic root motion was identical before and after the placement of coils and occluders (Table 3), thereby minimizing or completely avoiding any friction at the interface between occluder and biological tissue, and promoting instead the integration of coils and occluder into the healing process of the aortic tissue. The fundamental problem associated with the placement of a stent graft into the ascending aorta in dissection had been recognised previously [7,9,16] as any device would always be placed in diseased or even dissected tissue even if initial seal could be achieved; even with technological advances and a dedicated stent graft designed for the ascending aorta, its use in acute dissection was not approved (ARISE study and others). While stent grafts failed to portend a long-term success, they could at best be characterised as a temporizing solution for elective surgical conversion. Whether the concept of an endo-Bentall with an integrated aortic valve as an anker point (instead of landing a stent graft in diseased tissue) would solve the problem of stent-induced erosion in a mobile ascending aorta remains to be determined; today, this concept appears unlikely to be widely adopted as it comes with the sacrifice of the native aortic valve [14,17,18].

Although the early experience with the focal occlusion of entries in (essentially chronic) cases unfit for open surgery is promising, this “focal concept” targeting a mayor entry tear needs to be scrutinised in larger series or registries. So far, the experience is limited to a few patients, although with no failure yet, thus constituting a highly selective group of patients (or selection bias). Moreover, procedures were performed in a highly specialised centre by super-specialised operators, and yet, were also associated with a rather extensive radiation burden and duration; in addition, in the early stage of the learning curve, all procedures were performed under general anaesthesia, and thus needed streamlining. Nevertheless, new interventional approaches to address difficult scenarios in the setting of proximal dissection are feasible and should be documented and meticulously followed in international registries (as randomised trials are unlikely to materialise for various reasons).

With better diagnostics and initial management, the aortovascular community is likely to be seeing more cases of proximal aortic dissection that are not candidates of classic surgical aortic repair; demographic changes will also increase the number of patients for whom open surgery is no option. At the very least, experienced aortic centres with a multidisciplinary team approach should be open to new solutions for old problems; those places should feel the some responsibility to advance clinical research and create strategies in unchartered territories at best with a background of a profound understanding of disease and healing processes.

## 5. Limitations

This is a small retrospective cohort study that compares two different concepts, which of course need to be subjected to the scrutiny of a larger registry or even a randomised comparison (with a further improvement of the technologies used). Moreover, aortic root motion was analysed based on 2D DSA images rather than 4D MRI [19,20] or ECG-gated retrospective CT, which could be more accurate in a temporal–spatial tracking manner. 

## Figures and Tables

**Table 1:** Demographics and procedure details.

	Group I—Stent Graft				Group II—Occluding Device			
	Patient 1	Patient 2	Patient 3		Patient 4	Patient 5	Patient 6	
**Demographics**								
Age at procedure (years)	76	78	79	77.7 ± 1.53	75	85	78	79.3 ± 5.13
Gender	Female	Female	Male		Female	Female	Male	
Hypertension	Yes	No	Yes		Yes	No	Yes	
Diabetes	No	No	No		No	No	No	
Charlson comorbidity index	9	3	4		5	4	4	
EuroScore II	20.4	5.84	4.88		31.41	11.47	23.94	
**Procedure details**								
Total diameter of Asc. Ao. (mm)	55	50	66		66	62	42	
Max diameter of false lumen (mm)	29	25	55		46	53	10	
Number of entry tear(s)	1	1	1		1	1	1	
Devices	Zenith^®^ TX2^®^	Gore Tag^®^	Gore CTag active control		Coils + Amplatzer PFO occluder	Coils + Occlutech ASD occluder	Coils + Amplazter ASD occluder	
Device dimension (mm)	34 × 77	37 × 100	45 × 100		18	14	14	
General anaesthesia	Yes	Yes	Yes		Yes	Yes	Yes	
Procedure time (mins)	64	319	174	185 ± 127	180	157	120	152 ± 30
Total contrast (mL)	70	140	190	133 ± 60	60	140	170	123 ± 56
Total radiation dose (uGym^2^)	523.92	6289.09	17,901.52	8238 ± 8851	14,708.06	11,697.69	21,561.91	15,989 ± 5055
Procedure outcome	Successful	Successful	Unsuccessful		Successful	Successful	Successful	
Hospital stays (days)	9	6	19	11 ± 6	4	6	3	4 ± 1

**Table 2:** Follow-up details.

	Group I—Stent Graft			Group II—Occluding Device		
	Patient 1	Patient 2	Patient 3	Patient 4	Patient 5	Patient 6
**Medication**						
Beta-blocker	Bisoprolol	Bisoprolol	Bisoprolol	None	Bisoprolol	None
ACEi/ARB	None	None	None	None	Ramipril	Ramipril
CCB	Amlodipine	None	Amlodipine	Amlodipine	Amlodipine	None
Anticoagulant	None	None	Apixaban	None	Rivaroxaban	None
Antiplatelet	None	Aspirin	None	Aspirin	None	Aspirin
**Adverse event**						
Device-related complication	SINE	SINE	Migration	No	No	No
Re-intervention	No	No	Yes	No	No	No
**Survival**						
At 1 year	Yes	Yes	Yes	Yes	Yes	Yes
At 5 years	No	Yes	Yes	Yes	Yes	Yes
Follow-up duration (months)	18	86	47	79	76	72

**Table 3:** Maximum aortic root motion under aortogram and motion changed before and after procedure.

Displacement (mm)	Group I—Stent Graft			Group II—Occluding Device		
	Patient 1	Patient 2	Patient 3	Patient 4	Patient 5	Patient 6
Pre-procedure	6.84	8.51	6.78	5.59	3.23	13.3
Post-procedure	3.16	7.53	4.01	5.23	3.66	15.6
Motion changed	−53.8%	−11.5%	−40.9%	−6.41%	+13.2%	+17.4%

## Data Availability

The data presented in this study are available on request from the corresponding author.
